# Analysis and Prediction of Highly Effective Antiviral Peptides Based on Random Forests

**DOI:** 10.1371/journal.pone.0070166

**Published:** 2013-08-05

**Authors:** Kuan Y. Chang, Je-Ruei Yang

**Affiliations:** Department of Computer Science and Engineering, National Taiwan Ocean University, Keelung, Taiwan; Center for Genomic Regulation, Spain

## Abstract

The goal of this study was to examine and predict antiviral peptides. Although antiviral peptides hold great potential in antiviral drug discovery, little is done in antiviral peptide prediction. In this study, we demonstrate that a physicochemical model using random forests outperform in distinguishing antiviral peptides. On the experimental benchmark, our physicochemical model aided with aggregation and secondary structural features reaches 90% accuracy and 0.79 Matthew's correlation coefficient, which exceeds the previous models. The results suggest that aggregation could be an important feature for identifying antiviral peptides. In addition, our analysis reveals the characteristics of the antiviral peptides such as the importance of lysine and the abundance of α-helical secondary structures.

## Background

Antiviral peptides (AVPs) are an unconventional perspective for treating viral infections. Antiviral researches have undergone for more than half a century [1]–[3]. Although the traditional trial-and-error biochemical approach has led to the discovery of several antiviral nucleoside and non-nucleoside analogues such as brivudine against varicella-zoster virus [4], acyclovir against herpes simplex virus (HSV) [5], and azidothymidine (AZT) [6], stavudine [7]–[9] and efavirenz [10] against human immunodeficiency virus (HIV), the process is costly and time-consuming. Besides, severe toxicity is often a problem [11]. Instead, lower toxicity of antiviral peptides or proteins such as enfuvirtide against HIV virus [12] and DRACO [13], a potential panacea for all viruses, become an appealing alternative [14].

AVPs are known to fight against various viruses. All of the AVPs are derived from either synthetic combinatorial libraries or segments of natural proteins and their homologues. A list of highly effective antiviral peptides against HIV [15], HSV [16], hepatitis C virus [17], influenza virus [18]–[20], rabies virus [21], and west nile virus [22] has been compiled into an online database AVPpred [23]. Recently, there is an dedicated AVP database HIPdb for HIV, comprehensively collecting the experimentally validated HIV inhibiting peptides [24].

Several mechanisms are available for AVPs to fight against viruses. Antiviral therapeutics agents are known to block the attachment of viruses, prevent from the fusion of viruses to host cells, interrupt the signaling process of viruses, or inhibit the replication of viruses in host cells which may involve DNA polymerase, reverse transcriptase, integrase, and protease [14]. Currently studies have shown that AVPs inhibited the fusion of viruses to the cells [25], [26]; others have shown that AVPs interfered the replication of viruses [27]–[29].

Little is done in predicting and examining antiviral peptides. Broadly speaking, antiviral peptides should be a part of antimicrobial peptides, which fight against bacteria, fungi, parasites, and viruses. Several studies have been done in antimicrobial peptides [30]–[35], but a recent study by Thakur *et al.* demonstrated that antimicrobial peptide predictors are not suitable to assess AVPs [23]. In addition, this study was the first to explore four different approaches to predict effective AVPs: motif, sequence alignment, amino acid composition, and physicochemical features. Their results demonstrated that a support vector machine (SVM) approach using physiochemical features was a powerful method to identify AVPs. However, it is not clear whether key residues exist in AVPs and whether other methods can outperform SVM in predicting AVPs.

In this study, we demonstrate that our random forests (RF) model based on physiochemical properties works better for identifying AVPs. Physicochemical properties of peptides are a useful means to identify AVPs. A previous study demonstrated that predicting antimicrobial peptides (AMP) could depend on sequence-derived physicochemical properties and this study also suggested that aggregation could be important for classifying AMPs [33]; A recent study indeed showed that identifying AVPs using physicochemical properties of peptides worked [23]. Here we further investigated this finding.

## Materials and Methods

### Training, validation, and test data sets

The data sets were obtained from the study by Thakur *et al.* [23]. 1,056 peptides were validated experimentally, containing 604 highly effective AVPs and 452 non-effective peptides; another 604 peptides without experimental validation were non-effective from the study by Lata *et al.* [32]. Each of the peptides in the data sets was different from one another.

Two training sample sets and two independent test sets were established based on the data described above. Here we followed the same nomenclature used in the study by Thakur *et al.* [23]. 10-fold cross-validation was performed in our analysis, where the training and validation sets came from either of the two sample sets T^544P+407N^ and T^544P+544N*^. T^544P+407N^ consisted of 544 highly effective AVPs and 407 non-effective experimental peptides; T^544P+544N*^ contained the same 544 positive AVPs but different 544 non-experimental negative peptides. The independent test sets V^60P+45N^ and V^60P+60N*^ were used for the benchmark. V^60P+45N^ consisted of 60 highly effective AVPs and 45 non-effective peptides; V^60P+60N*^ contained 60 positive peptides and 60 non-experimental negative peptides.

### Viral proteomes

The viral proteins were obtained from the viral genome database at the NCBI Entrez (January 2013), which consisted of 3251 viral genomes and viroids. All the 41316 viral proteins expressed by these genomes and viroids were retrieved. Non-standard amino acids such as ‘B’, ‘J’, ‘U’ and ‘X’ in the viral proteins, which were less than 0.01% of the overall residues, were eliminated during the analysis. The viral proteomes were treated as non-effective antiviral peptides under a simple assumption that the viral proteins would not discourage themselves to develop.

### RF classifier

RF is a classification method using an ensemble of unpruned decision trees with randomly selected features. The RF algorithm integrates random subspace method [36] into the concept of bootstrap aggregating or bagging [37] to generate an ensemble of decision trees. Independently each decision tree is best split by a small fraction of randomly selected features, trained on cases chosen by random sampling with replacement to all the available data. The aggregated decision trees then determine which class the predicted case belongs. The RF classifier known to avoid overfitting is a highly accurate method in many classification problems [38], [39]. In this study, the randomForest R package version 4.6–7 was utilized [40], which was based on Breiman and Cutler's algorithm [39]. Two parameters the number of *ntree* decision trees and the number of *mtry* selected features were set as follows: *ntree*  = 100 and 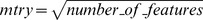 as recommended [39]. One additional advantage of the RF model is that the model is possible to interpret the importance of the features using measures such as decrease mean accuracy or Gini importance.

### Artificial Neural Network (ANN) classifier

In this study, ANN was trained by the backpropagation algorithm. Its learning rate and momentum rate were equal to 0.3 and 0.2 respectively. The number of hidden units was set to half of the number of features and the number of classes.

### Linear Discriminant Analysis (LDA) classifier

The MASS R package version 7.3–26 was utilized to build the LDA models in this study. The LDA models seek the best linear combination of the features to separate AVPs from others.

### Gini importance

Gini importance or the mean decrease of Gini index (MDGI) is a robust quantity to measure variable importance in the RF model [41]. Gini index is an impurity quantity defined as follows:
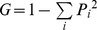
where *i* contains all the classes and *P_i_* is the fraction of class *i*. In our case there are two classes: AVPs and non-AVPs. The Gini index ranges from 0 to 1 and the index is closer to 0 if the sample is purer. The decrease of Gini index in a tree model refers to the difference between the Gini index of a parent node and the weighted Gini index of its descending nodes. Given a particular variable, the MDGI is the sum of the decrease of Gini index over the number of the decision trees in the RF. The larger the MDGI is, the more important the variable is.

### Amino Acid Composition (AAC)

Amino acid composition is the ratio of each amino acid in a peptide. The ratio of an amino acid with type *T* in a peptide X is calculated as follows:
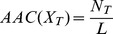
where N_T_ is the number of the amino acid with type *T* and *L* is the length of peptide X.

### Physicochemical properties

In the first attempt, 544 physicochemical indices in amino acid index database (AAindex version 9.1) [42] were examined. Each index contains 20 numerical values to represent the specific physicochemical properties of 20 amino acids. A simple linear addition model, the scalar product of each of the 544 indices and each of the amino acid composition of peptides, was used for the evaluation. Since the number of the parameters generated by these 544 indices was much larger than the number of the training cases, identifying crucial indices using feature selection was recommended. We tested few feature selection methods such as minimum Redundancy Maximum Relevance to identify the crucial indices [43]. However, neither the top-ranked indices from our study nor those by Thakur *et al.* [23] provided a satisfactory predictive performance in the RF models. In fact, our results suggested that the RF models with more indices performed worst (data not shown), which followed similar trends as indicated in the previous finding [34].

In the next attempt, the following physicochemical properties were selected: length, net charges, instability index, aliphatic index, and hydropathicity. Our basic physicochemical models were built by utilizing the five properties combined with amino acid composition. The advanced features such as secondary structures of peptides described below were considered.

The instability index, an estimate of peptide stability, is calculated as follows:
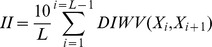
where *L* is the length of peptide and *DIWV* from the study by Guruprasad *et al.* is an instability weight value of a dipeptide starting at position i [44]. Peptides with II values greater than 40 are considered to be unstable.

The aliphatic index, the relative volume of aliphatic residues in a peptide, is calculated as follows:
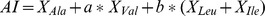
where *a* and *b* are the constants, which represent the relative volume of valine and leucine or isoleucine to alanine. X_Ala_, X_Val_, X_Leu_, and X_Ile_ are the fractions of alanine, valine, leucine and isoleucine multiplied by 100, respectively [45].

Grand average of hydropathicity index (GRAVY) is used to represent the hydrophobicity value of a peptide, which calculates the sum of the hydropathy values of all the amino acids divided by the sequence length. GRAVY was calculated using the hydropathy values from Kyte and Doolittle [46]. Positive GRAVY values indicate hydrophobic; negative values mean hydrophilic. All these physicochemical values could be obtained directly from the ExPASy website (http://www.expasy.org) [47].

### Aggregation

AGGRESCAN was utilized to estimate the aggregation tendencies of a peptide [48]. AGGRESCAN, which applied aggregation propensities of amino acids derived from the experimental data of β-amyloid peptides, is a good indicator of *in vivo* aggregation.

### Secondary Structure

Protein secondary structure prediction (PSSpred version 2.0), which was a neural network classifier integrated into the famous I-TASSER server [49], was utilized to predict the secondary structure of a peptide [50]. Each amino acid on the peptide was classified into α-helix, β-sheet, or random coil. The number of each type of secondary structure was then recorded.

### Validation

The performance characteristics of the models such as sensitivity, specificity, accuracy, and MCC are defined as follows:
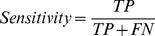


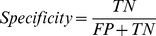


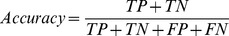


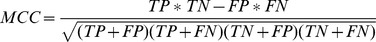



TP, TN, FP, and FN represent true positives, true negatives, false positives, and false negative, respectively. Sensitivity, specificity, and accuracy are the percentage of the correct predictions on positive data, negative data, and all of the data, respectively. MCC is used to evaluate the performance of the binary classifier. It works even when the sizes of classification classes differ. The value of MCC is between −1 and 1. The larger the MCC value, the better the classifier.

## Results

### Amino acid composition of the AVPs

**Figure 1:**
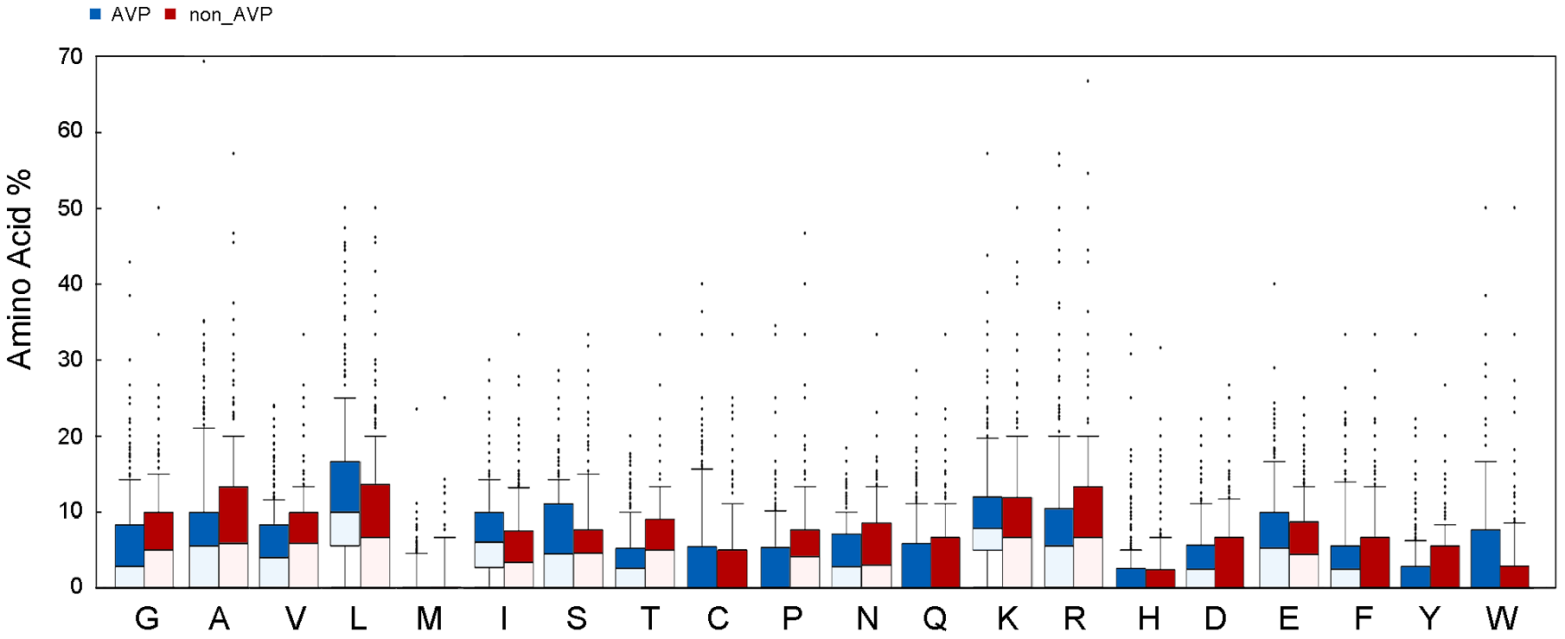
Statistical distribution of the amino acid composition of AVPs and non-AVPs. The blue and red bars represent the amino acid composition of 604 antiviral peptides and 452 non-antiviral peptides.

**Figure 2:**
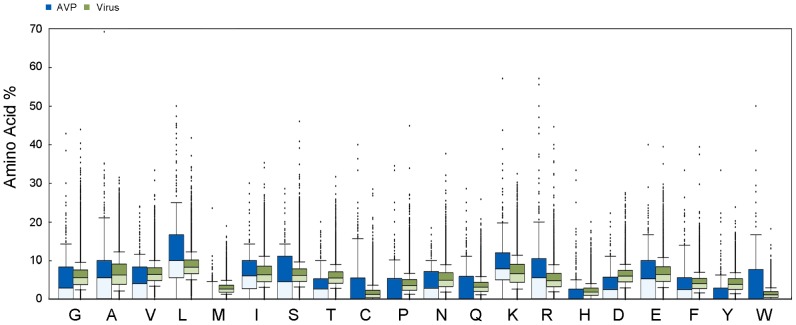
Statistical distribution of the amino acid composition of AVPs and viral proteomes. The blue and green bars represent the amino acid composition of 604 antiviral peptides and 41316 viral proteins.

The comparison of amino acid composition of AVPs was shown in Fig. 1 and Fig. 2. The viral proteomes were chosen to be an optional baseline. Compared to the non-effective experimental peptides and the viral proteomes, the AVPs consistently showed higher percentages of leucine and lysine, but lower percentages of threonin, proline, and valine. Leucine, medium-sized hydrophobic residue, was the most abundant residues in the AVPs. In addition, a clear preference was shown for a basic residue lysine, which was also known as the key characteristics for anti-microbial peptides.

### Physicochemical properties, aggregation, and secondary structures of the AVPs

**Figure 3:**
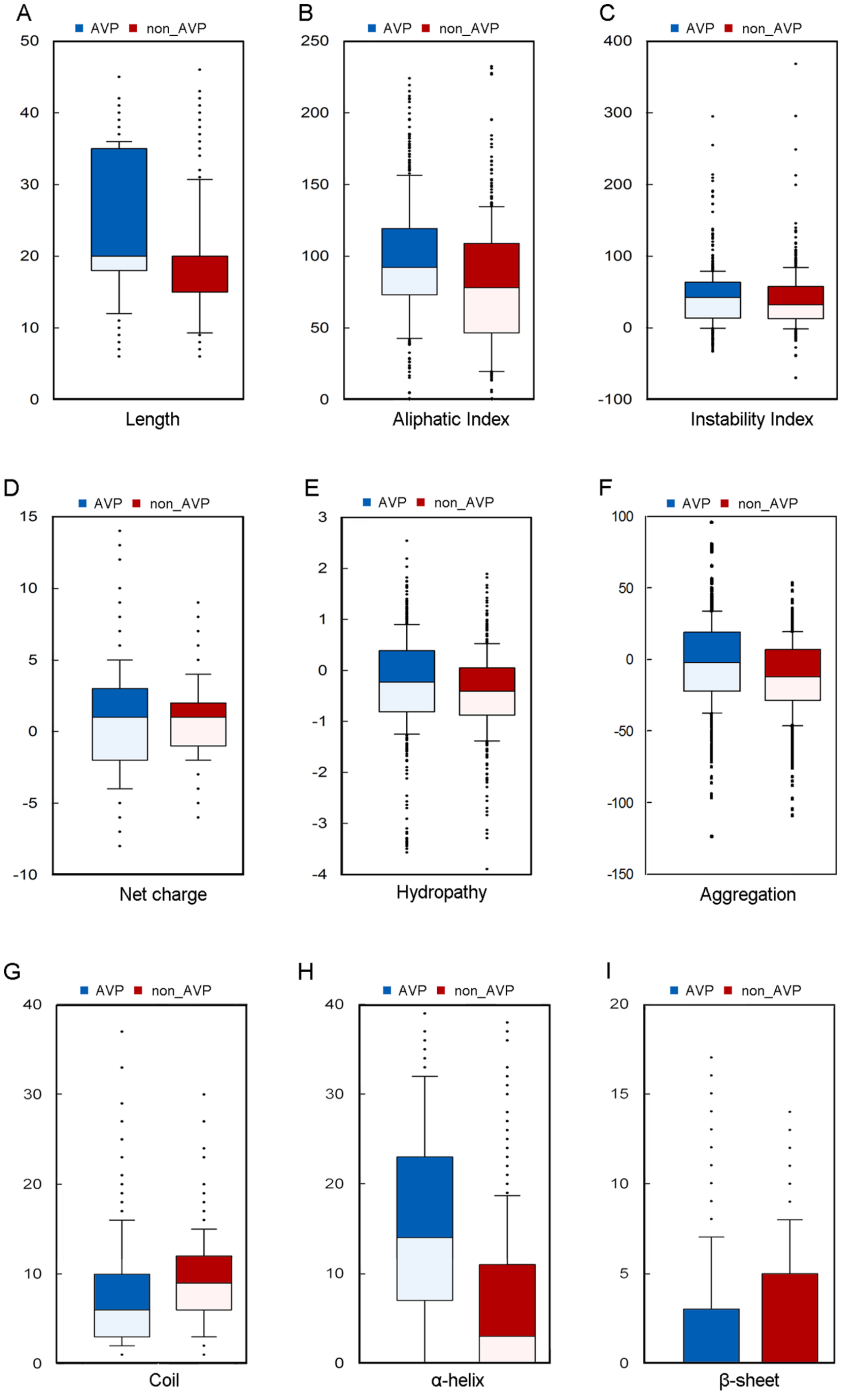
Statistical distribution of the physicochemical properties of AVPs and non-AVPs. 604 antiviral peptides and 452 non-antiviral peptides in the AVPpred study are compared, including (A) length, (B) aliphatic index, (C) instability index, (D) net charge, (E) hydropathy, (F) aggregation, (G) random coil, (H) α-helix, and (I) β-sheet.

**Figure 4:**
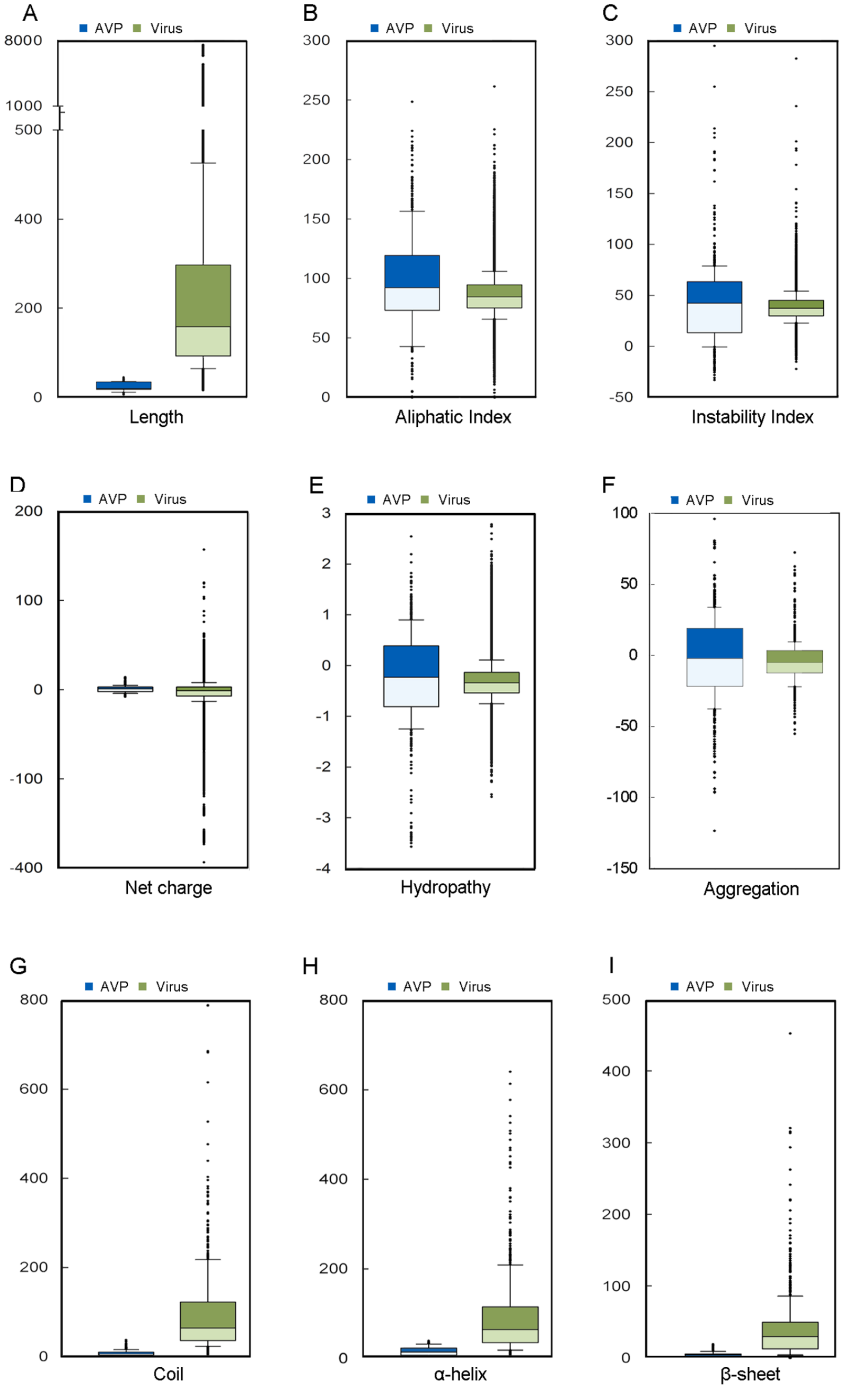
Statistical distribution of the physicochemical properties of AVPs and viral proteomes. 604 antiviral peptides and 41316 viral proteins are compared, including (A) length, (B) aliphatic index, (C) instability index, (D) net charge, (E) hydropathy, (F) aggregation, (G) random coil, (H) α-helix, and (I) β-sheet. Due to intense computing, the viral data at (F-I) were based on randomly selected 604 proteins instead of 41316 viral proteins.

The statistical analysis of the physicochemical properties of the AVPs was done (Fig. 3 and Fig. 4). The following properties were examined, including length, aliphatic index, instability, net charge, hydropathy, aggregation, and secondary structure. The lengths of the 604 AVPs ranged from 6 to 45 amino-acid residue, with an average length of 24.2 residues; the 452 non-effective AVPs had similar range with an average length of 18.2 residues; the viral proteins were longer with an average length over 200 residues. The AVPs had the strongest aliphatic tendency among them all (Fig. 3B and Fig. 4B). Indeed, the AVPs had the largest portions of aliphatic residues such as leucine, isoleucine, and valine (Fig. 1 and Fig. 2). Besides, only the AVPs had an average instability value over 40, which considered to be unstable; both the non-effective AVPs and viral proteins had an instability value less than 40 (Fig. 3C and Fig. 4C). In terms of residue charge, the AVPs had higher tendency to be charged positively than the non-effective peptides; the viral proteins preferred negative net charges (Fig. 3D and Fig 4D). The AVPs had a slightly negative GRAVY score, but higher than both the non-effective peptides and the viral proteins (Fig. 3E and Fig. 4E). In the aggregation analysis, the AVP also had a slightly negative AGGRESCAN score on average, but higher than the non-effective peptides and the viral proteins (Fig. 3F and Fig 4F).

Each residue of the peptides was classified into α-helix, β-sheet, or coil according to the secondary structure prediction. More than half of all the residues of the AVPs were α-helix (Fig. 3H), but the AVPs had lower portions of coil than the non-effective peptides and the viral proteins (Fig. 3G and Fig. 4G). β-sheet propensity was less clear, for few residues were classified into β-sheet (Fig. 3I and Fig 4I).

### Feature importance of amino acid composition

**Figure 5:**
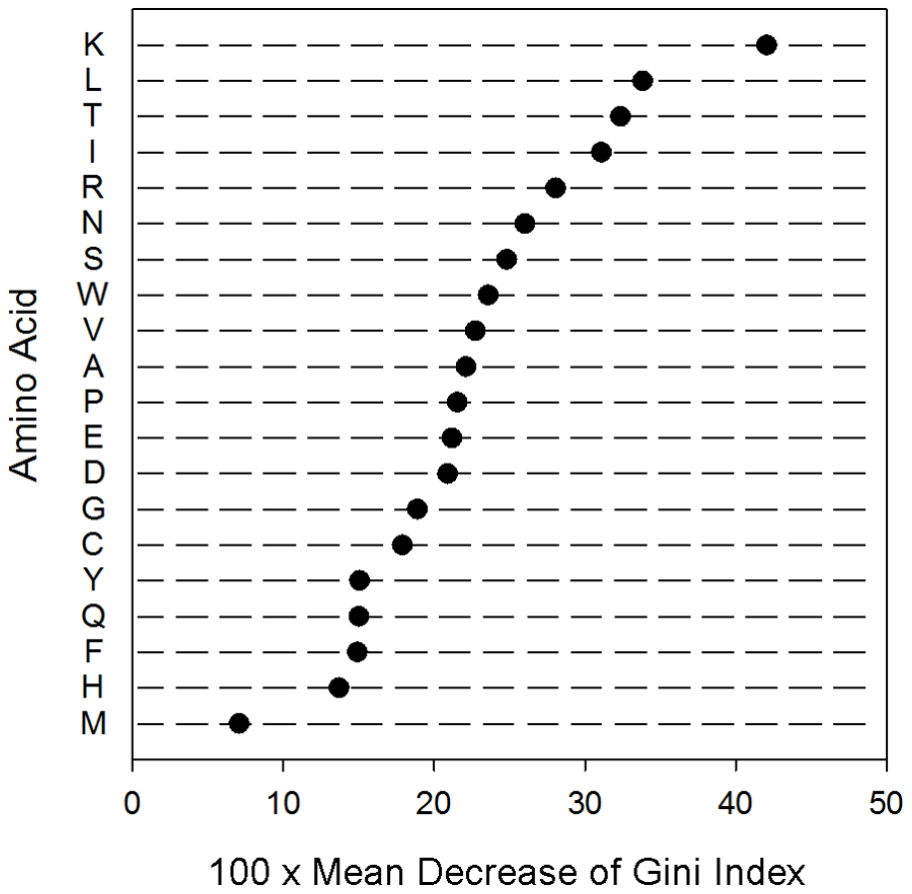
Feature importance of amino acid composition. The importance of each amino acid is measured using the mean decrease of Gini index (MDGI) of the 20 amino acids ranked by the RF model built for the AVPs and non-AVPs. The larger the MDGI value, the more important the residue.

The analysis of Gini importance on the RF model built with amino acid composition was performed. Lysine, a positively charged molecule, was the most important residue among the 20 amino acids for classifying AVPs. The result was consistent with those evaluated by different methods, which ranked the importance of amino acid similarly albeit slightly different (Table S1). Medium-size hydrophobic residues such as leucine and isoleucine also played a role in distinguishing AVPs from non-AVPs. Besides, the importance of threonine was also identified (Fig. 5).

In addition, the same Gini analysis of a RF model on an AMP database was performed. Although both lysine and arginine were abundant in the AMPs, the Gini analysis showed that neither lysine nor arginine was the most effective residue to tell apart AMPs from non-AMPs (Fig. S1). Our results indicated relatively less abundant residues in the AMPs such as methionine, aspartic acid, glutamic acid, and threonine are important to distinguish AMPs (Fig. S1). This differs from what we have seen in the AVPs, where lysine was the most effective residue.

### Performance analysis on the training samples

All eight RF models were examined using 10-fold cross-validation trained by either T^544P+407N^ or T^544P+544N*^ as summarized in Table 1. Those models trained by T^544P+544N*^ were marked with the number sign #; those trained by T^544P+407N^ were without the sign. In order to easily compare AVPpred, our models named similarly after the models of AVPpred. The abbreviations compo, physico, structure, and agg represented amino acid composition, the amino acid composition plus five basic physicochemical properties, secondary structure, and aggregation respectively. For example, RFphysico was a RF model based on five basic physicochemical properties and amino acid composition. RFcompo+structure+agg was based on amino acid composition, secondary structure, and aggregation.

**Table 1:** Performance of the random forests models using 10-fold cross-validation.

Data	Model	Sensi tivity	Speci ficity	Accu racy	MCC
T^544P+407N^	RFcompo	85.3	82.6	84.1	0.68
	RFcompo+agg	86.4	82.8	84.9	0.69
	RFcompo+structure	86.8	81.8	84.6	0.69
	RFcompo+structure +agg	86.6	83.0	85.1	0.70
	RFphysico	85.9	81.8	84.2	0.69
	RFphysico+agg	85.5	81.6	83.8	0.67
	RFphysico+structure	86.6	82.1	84.6	0.69
	RFphysico+structure +agg	86.6	81.6	84.4	0.68
T^544P+544N*^	RFcompo^#^	89.5	92.6	91.1	0.82
	RFcompo+agg^#^	89.5	92.6	91.1	0.82
	RFcompo+structure^#^	89.0	94.1	91.5	0.83
	RFcompo+structure +agg^#^	89.0	94.1	91.5	0.83
	RFphysico^#^	88.1	93.8	90.0	0.82
	RFphysico+agg^#^	88.1	93.8	90.0	0.82
	RFphysico+structure^#^	89.0	93.9	91.5	0.83
	RFphysico+structure +agg^#^	89.0	93.9	91.5	0.83

T^544P+544N*^ contained non-experimental peptides. The models trained by T^544P+544N*^ were marked by the number sign #.

In the 10-fold cross-validation, the performances of the eight RF models were close. In the T^544P+407N^ training data, the best model RFcompo+structure+agg had an accuracy of 92% and 0.83 MCC. Both RFcompo+structure and RFphysico+structure reached a slightly higher accuracy of 85% and 0.69 MCC than RFcompo and RFphysico. In the T^544P+544N*^ training data, similar trends were found. Four models RFcompo+structure#, RFcompo+structure+agg#, RFphysico+structure#, and RFphysico+structure+agg# achieved a high accuracy of 92% and 0.83 MCC. As suggested before, different levels of the performances on the two training data sets might be due to T^544P+544N*^ containing non-experimental peptides [23]. Those non-experimental peptides were retrieved from non-secreted proteins, for the original study assumed antimicrobial peptides to be naturally secreted proteins [32]. The non-secreted proteins might be very different from the AVPs, thus distinguishing AVPs from those non-experimental peptides would be easy.

### Performance evaluation on the independent test sets

Table 2 shows the performances of four learning methods with the standard models. The models using ANN, LDA, and RF were built on the same datasets. Two best SVM models of AVPpred, the only other tool available for predicting AVPs, were also included. All these models were evaluated on the two test sets V^60P+45N^ and V^60P+60N*^. V^60P+45N^ had only experimentally verified peptides, but V^60P+60N*^ contained non-experimental peptides. As seen in Table 2, the LDA models performed fairly, the ANN models gave a satisfactory prediction, and the SVM or RF models were very good in distinguishing AVPs. In general, the comparison results demonstrated that the RF models were superior to the other models in this problem.

**Table 2:** Performance of the standard models by different learning methods on validation sets V^60P+45N^ and V^60P+60N*^.

Data	Model	Sensitivity	Specificity	Accuracy	MCC
V^60P+45N^	AVPcompo	83.3	**88.9**	85.7	0.72
	AVPphysico	88.3	82.2	85.7	0.71
	ANNcompo	76.7	75.6	76.2	0.52
	ANNphysico	85.0	80.0	82.9	0.65
	LDAcompo	85.0	44.4	67.6	0.33
	LDAphysico	88.3	53.3	73.3	0.45
	RFcompo	86.7	80.0	83.8	0.67
	RFphysico	**93.3**	77.8	**86.7**	**0.73**
V^60P+60N*^	AVPcompo^#^	83.3	**98.3**	90.8	0.83
	AVPphysico^#^	**93.3**	91.7	92.5	0.85
	ANNcompo^#^	81.7	93.3	87.5	0.76
	ANNphysico^#^	91.7	90.0	90.8	0.82
	LDAcompo^#^	78.3	66.7	72.5	0.45
	LDAphysico^#^	81.7	75.0	78.3	0.57
	RFcompo^#^	**93.3**	93.3	**93.3**	**0.87**
	RFphysico^#^	90.0	95.0	92.5	0.85

V60P+60N^*^ contained non-experimental peptides. The models trained by T544P+544N^*^ were marked by the number sign #; the models trained by T544P+407N had no marks. All the AVP models here were built using SVM [23].

On the V^60P+45N^ test set, the RF models performed well (Table 2). The best RF models consistently outperformed the best models of AVPpred. For example, RFphysico surpassed AVPphysico, which was the performance indicator of AVPpred with an accuracy of 86% and 0.71 MCC, while both models utilized only 25 physicochemical properties.

On the V^60P+60N*^ test set containing non-experimental peptides, similar results were seen. For this test set, the two best models of AVPpred trained by T^544P+544N*^, AVPcompo# and AVPphysico#, were included. Our best model RFcompo# achieved the maximal accuracy of 93% and the highest 0.87 MCC. RFcompo# outperformed AVPcompo# while RFphysico and AVPphysico were comparable.

Whether additional features such as aggregation and secondary structure could improve the prediction was examined. Table 3 shows a performance comparison among the eight RF models. Generally speaking, adding aggregation to the RF models tended to improve the performance slightly. For example, on both test sets, RFcompo+agg surpassed RFcompo while RFcompo+agg# and RFcompo# were comparable. Adding secondary structure could also improve the RF models. For example, RFcompo+structure outperformed RFcompo. In addition, on the V^60P+45N^ test set, our best model RFcompo+structure+agg achieved the maximal accuracy of 90% and the highest 0.79 MCC. On the V^60P+60N*^ test set, our best model RFcompo# and RFcompo+agg# achieved the maximal accuracy of 93% and the highest 0.87 MCC. The overall results demonstrated that these features could be useful for identifying the AVPs.

**Table 3:** Performance of the random forests models on validation sets V^60P+45N^ and V^60P+60N*^.

Data	Model	Sensi tivity	Speci ficity	Accu racy	MCC
V^60P+45N^	RFcompo	86.7	80.0	83.8	0.67
	RFcompo+agg	88.3	82.2	85.7	0.71
	RFcompo+structure	90.0	86.7	88.6	0.77
	RFcompo+structure +agg	91.7	86.7	**89.5**	**0.79**
	RFcompo^#^	93.3	48.9	74.3	0.48
	RFcompo+agg^#^	91.7	51.1	74.3	0.48
	RFcompo+structure^#^	93.3	42.2	71.4	0.43
	RFcompo+structure +agg^#^	93.3	48.9	74.3	0.48
	RFphysico	93.3	77.8	86.7	0.73
	RFphysico+agg	90.0	82.2	86.7	0.73
	RFphysico+structure	91.7	82.2	87.6	0.75
	RFphysico+structure +agg	91.7	82.2	87.6	0.75
	RFphysico^#^	90.0	53.3	74.3	0.48
	RFphysico+agg^#^	88.3	53.3	73.3	0.45
	RFphysico+structure^#^	90.0	40.0	68.6	0.35
	RFphysico+structure +agg^#^	**95.0**	48.9	75.2	0.51
V^60P+60N*^	RFcompo	86.7	56.7	71.7	0.45
	RFcompo+agg	88.3	56.7	72.5	0.47
	RFcompo+structure	90.0	60.0	75.0	0.52
	RFcompo+structure +agg	91.7	56.7	74.2	0.52
	RFcompo^#^	93.3	93.3	**93.3**	**0.87**
	RFcompo+agg^#^	91.7	**95.0**	**93.3**	**0.87**
	RFcompo+structure^#^	93.3	88.3	90.8	0.82
	RFcompo+structure +agg^#^	93.3	86.7	90.0	0.80
	RFphysico	91.7	41.7	66.7	0.39
	RFphysico+agg	90.0	45.0	67.5	0.39
	RFphysico+structure	91.7	48.3	70.0	0.44
	RFphysico+structure +agg	91.7	48.3	70.0	0.44
	RFphysico^#^	90.0	95.0	92.5	0.85
	RFphysico+agg^#^	88.3	91.7	90.0	0.80
	RFphysico+structure^#^	90.0	88.3	89.2	0.78
	RFphysico+structure +agg^#^	**95.0**	86.7	90.8	0.82

V^60P+60^N^*^ contained non-experimental peptides. The models trained by T^544P+544^N^*^ were marked by the number sign #; the models trained by T^544P+407^N had no marks.

## Discussion

This was the first study to apply RF into AVP prediction. Our RF models were based on size, amino acid composition, net charge, aliphaticity, instability, hydrophobicity, and secondary structure. The results demonstrated that predicting AVPs by the RF models through the basic physicochemical properties worked. On the independent test data provided by AVPpred, our evaluation indicated that RF worked better than SVM in distinguishing AVPs using these physiochemical properties. Our result supports that RF, a robust classifier, excels in many problems [38].

In order to reach optimal performances, several training designs for the RF models were explored. One option was to remove highly similar sequences from the training cases. The performance dropped as the RF models were trained by the non-redundant cases, which were generated by removing highly similar cases over 80% identities, leaving out more than one-third of the entire training cases. Additional analyses on the model attributes were also done such as increasing the number of attributes by AAindex and replacing the secondary-structure counts with the secondary-structure percentages. However, none enhanced performance. This suggests that improving AVP prediction is not a trivial task.

To compare AVPpred fully, the effects of different training sets on the RF models were examined. One set had the experimental data; the other contained the negatives without experimental verification. This led to different levels of specificity. Since true negatives in biological context are often limited and difficult to obtain, hypothetical negatives or negatives without experimental verification are the substitutes. However, ideally all true negatives for building the models should be verified experimentally to avoid performance errors. More attention, therefore, should be paid to the models with the experimental data. Our RF models outperformed the previous ones in either training set, but more obvious in the experimental training data.

Feature importance of amino acid composition of the AVPs was analyzed using the experimental data. Lysine, also known as a key residue in antimicrobial peptides, was the most important residue in distinguish AVPs. The residues with great importance need not to be the abundant residues. Our analyses showed that the AVPs were abundant in leucine, lysine, alanine, and glutamic acid (Fig. 1 and Fig. 2), which supports the previous finding by HIPdb [24]. Several other interesting properties were found in our analysis, but we only emphasized lysine, for this finding was supported by various feature ranking methods. However, why the AVPs were biased toward lysine is not clear. One possible explanation is that the positively charged AVPs might interact with enveloped viruses like HIV [30], inhibiting the entry of the viruses into the cells.

The aggregation propensities of the AVPs were revealed. Our results suggested that the AVPs had higher tendencies to be aggregated *in vivo* than the non-effective peptides and the viral proteins. Our RF models also indicated that aggregation could be an important feature for classifying the AVPs. It is not clear how aggregation affects the AVPs to restrain the viruses. However, aggregation has been suggested to be an important feature of AMPs, for accumulating and clumping peptides together could affect the peptide availability [33].

In addition, the abundance of α-helix secondary structures in the AVPs was discovered in this study. What role the secondary-structure preference plays in fighting viral infection is not understood. It has been suggested that the α-helical structures are an common element for protein-protein interactions [51] and the α-helical structures of antimicrobial peptides are linked to the permeability to the membranes to abrupt the cell membranes of bacteria [35]. Whether the α-helical structures of the AVPs, a subset of antimicrobial peptides, interact with the enzymes in the virus replication or have similar impact on host cell membranes still need to be investigated. However, given secondary structure information, the performance of the RF models could further improve. On the experimental benchmark, our model trained by amino acid composition, aggregation, and secondary structure achieved the optimal performances. The importance of secondary structure was also supported by the previous study–several top physiochemical indices were related to secondary structure [23].

## Supporting Information

Figure S1 Feature importance of amino acid composition of AMPs. The importance of each amino acid is measured using the mean decrease of Gini index (MDGI) of the 20 amino acids ranked by the RF model built for the AMPs and non-AMPs. The larger the MDGI value, the more important the residue.
(TIF)

Table S1 Feature importance of amino acid composition measured by different methods.
(DOCX)

Text S1 Supplementary Information for Figure S1.
(DOCX)
