# Supplementary figures and images for: Analysis and Prediction of Highly Effective Antiviral Peptides Based on Random Forests
Source: PLoS One. 2013 Aug 5;8(8):e70166. doi: 10.1371/journal.pone.0070166 (PMC3734225; doi:10.1371/journal.pone.0070166)

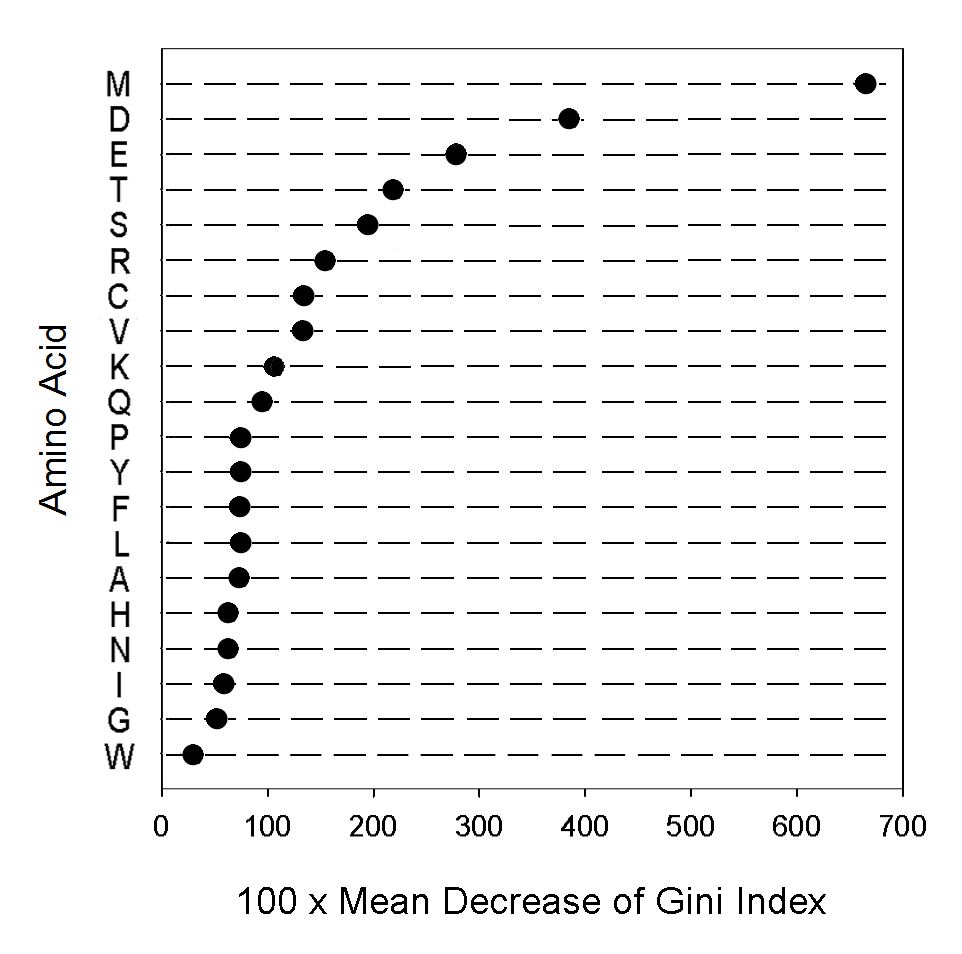

Supplement: Figure S1 — Feature importance of amino acid composition of AMPs. The importance of each amino acid is measured using the mean decrease of Gini index (MDGI) of the 20 amino acids ranked by the RF model built for the AMPs and non-AMPs. The larger the MDGI value, the more important the residue. (TIF) [file pone.0070166.s001.tif]
