# Supplementary material for: Analysis and Prediction of Highly Effective Antiviral Peptides Based on Random Forests
Source: PLoS One. 2013 Aug 5;8(8):e70166. doi: 10.1371/journal.pone.0070166 (PMC3734225; doi:10.1371/journal.pone.0070166)
Supplement: Table S1 — Feature importance of amino acid composition measured by different methods. (DOCX) [file pone.0070166.s002.docx]

**Table S1. Feature importance of amino acid composition measured by different methods.**

| Residue | Mean Decrease Gini Index | Mean Decrease Accuracy | MaxRel | mRMR |
| --- | --- | --- | --- | --- |
| A | 22.120 | 32.313 | 0.130 | -0.624 |
| C | 17.887 | 31.053 | 0.064 | -0.317 |
| D | 20.896 | 35.445 | 0.135 | -0.400 |
| E | 21.184 | 35.306 | 0.121 | -0.597 |
| F | 14.909 | 24.502 | 0.071 | -0.444 |
| G | 18.888 | 26.949 | 0.077 | -0.526 |
| H | 13.705 | 24.774 | 0.080 | -0.277 |
| I | 31.080 | 42.050 | 0.133 | -0.576 |
| K | 42.067 | 46.696 | 0.187 | 0.187 |
| L | 33.819 | 42.034 | 0.163 | -0.742 |
| M | 7.070 | 14.808 | 0.000 | -0.298 |
| N | 26.014 | 40.431 | 0.126 | -0.375 |
| P | 21.532 | 33.157 | 0.078 | -0.396 |
| Q | 15.015 | 29.442 | 0.070 | -0.439 |
| R | 28.053 | 35.961 | 0.146 | -0.560 |
| S | 24.808 | 36.576 | 0.126 | -0.468 |
| T | 32.348 | 39.922 | 0.114 | -0.456 |
| V | 22.736 | 30.073 | 0.110 | -0.537 |
| W | 23.587 | 37.702 | 0.098 | -0.332 |
| Y | 15.067 | 23.303 | 0.091 | -0.318 |
