# Supplementary material for: Analysis and Prediction of Highly Effective Antiviral Peptides Based on Random Forests
Source: PLoS One. 2013 Aug 5;8(8):e70166. doi: 10.1371/journal.pone.0070166 (PMC3734225; doi:10.1371/journal.pone.0070166)
Supplement: Text S1 — Supplementary Information for Figure S1. (DOCX) [file pone.0070166.s003.docx]

Supporting Information

We have performed the Gini analysis of the AMPs. The AMP and non-AMP data were obtained using a similar way to others [33, 35]. 1,893 AMPs were obtained from CAMP. 7,058 protein sequences from the reviewed UniRef50 of the Uniprot database were treated as non-AMPs, which were selected to be no more than 100 amino acids long and without antimicrobial annotation. The results indicated relatively less abundant residues in the AMPs such as methionine, aspartic acid, glutamic acid, and threonine are important to distinguish AMPs (Fig. S1).
